# Selections

**Published:** 1894-10

**Authors:** 


### Selections.

### GUAIACOL.

At a recent meeting of the Paris Society of Biology, a report of which we find in the *Union Médicale* for November 30, M. Gilbert and M. L. Maurat presented a communication in which it was remarked that guaiacol was one of the derivatives of creosote that had been most experimented with. The liquid guaiacol found in commerce, the authors went on to say, meaning the guaiacol obtained from creosote, was far from being almost a chemically pure product. It was a mixture of cresylol, guaiacol, and creosote in varying proportions; it might contain fifty per cent. of guaiacol, but sometimes it contained no more than twenty or even ten per cent. Pure guaiacol occurred in the form of hard, white rhomboidal crystals, almost insoluble in water, but soluble in alcohol, in oil, and in anhydrous glycerin. The taste of guaiacol is slightly sugary, with a pungent and burning after-taste. Pure guaiacol has been prepared synthetically by Behel and Chouy, and it was this product that the authors had used in their experiments. The amount of guaiacol, dissolved in glycerin or olive oil, necessary to kill a guinea-pig weighing a kilogramme is between five and fifteen grains, given hypodermically; it takes twenty-three grains given by the mouth to produce the same result. With a liquid guaiacol obtained from Germany, containing forty-six per cent. of guaiacol, 3.6 per cent of cresylol, and 50.5 per cent. of cresol and homocresol, from fifteen to twenty grains were found necessary to kill a guinea-pig weighing a kilogramme. This product, therefore, is less poisonous than pure guaiacol, but it contains less than half of the active principle. The chief effects of poisoning with guaiacol are agitation and then enfeeblement with retardation of the heart's action and of the breathing. Most of the secretions are increased in quantity, especially the lachrymal secretion. At the moment of death, which occurs during coma, the thermometer may fall as low as 68° F. [*jusqu'à* 20°]. The biological effects of liquid guaiacol resemble those of solid guaiacol, but the reduction of temperature and the increase of the secretions are less pronounced. The authors have used synthetic guaiacol for several months in a certain number of cases of consumption in various stages. They have given it in daily quantities of from six to eighteen grains, in the form of pearls, each containing three grains of the active principle in oily solution. As a general thing, the stomach bears the drug well if it is taken immediately before eating. Sometimes large doses cause vomiting, but this may be avoided easily by testing the patient's susceptibility by administering progressively increasing doses of the synthetic preparation.—*New York Medical Journal.*

